# *De novo* Assembly of the *Brugia malayi* Genome Using Long Reads from a Single MinION Flowcell

**DOI:** 10.1038/s41598-019-55908-y

**Published:** 2019-12-20

**Authors:** Joseph R. Fauver, John Martin, Gary J. Weil, Makedonka Mitreva, Peter U. Fischer

**Affiliations:** 1 Division of Infectious Diseases, Department of Medicine, Washington University School of Medicine, St. Louis, MO United States; 2 McDonnell Genome Institute, Washington University School of Medicine, St. Louis, MO United States; 3 Present Address: Department of Epidemiology of Microbial Diseases, Yale School of Public Health, New Haven, CT United States

**Keywords:** Medical genomics, DNA sequencing

## Abstract

Filarial nematode infections cause a substantial global disease burden. Genomic studies of filarial worms can improve our understanding of their biology and epidemiology. However, genomic information from field isolates is limited and available reference genomes are often discontinuous. Single molecule sequencing technologies can reduce the cost of genome sequencing and long reads produced from these devices can improve the contiguity and completeness of genome assemblies. In addition, these new technologies can make generation and analysis of large numbers of field isolates feasible. In this study, we assessed the performance of the Oxford Nanopore Technologies MinION for sequencing and assembling the genome of *Brugia malayi*, a human parasite widely used in filariasis research. Using data from a single MinION flowcell, a 90.3 Mb nuclear genome was assembled into 202 contigs with an N50 of 2.4 Mb. This assembly covered 96.9% of the well-defined *B. malayi* reference genome with 99.2% identity. The complete mitochondrial genome was obtained with individual reads and the nearly complete genome of the endosymbiotic bacteria *Wolbachia* was assembled alongside the nuclear genome. Long-read data from the MinION produced an assembly that approached the quality of a well-established reference genome using comparably fewer resources.

## Introduction

Lymphatic filariasis is one of the world’s leading causes of morbidity and disability adjusted life years, particularly in the low and middle-income countries of the tropics^1^. Lymphatic filariasis can cause severe swelling in limbs and the groin that can result in pain, disability, and social stigma. The most effective strategy for preventing transmission is community directed mass drug administration (MDA) of anti-helminthic drugs^2^. Currently, MDA is the foundation of the Global Program to Eliminate Lymphatic Filariasis (GPELF), a large-scale global health program aimed at eliminating lymphatic filariasis as a public health problem^2^. Since the initiation of this program, more than 7 billion treatments have been distributed which has reduced the population at risk of infection by 1/3^rd^ to an estimated 554 million people^3^. Lymphatic filariasis is caused by infection with the filarial nematodes *Brugia malayi*, *Brugia timori*, and *Wuchereria bancrofti*, the latter of which is the most common species^4^. These worms are transmitted to humans via the bite of infectious mosquitoes. A highly inbred *B. malayi* strain (FR3) maintained in gerbils (*Meriones unquiculatus*) is a widely used laboratory model to study lymphatic filariasis. The FR3 strain of *B. malayi* was used for gene discovery experiments in the Filarial Genome Project as early as 1994^5^. Whole genome sequencing and assembly of the *B. malayi* genome was reported in 2007 using a variety of approaches that included sequencing of bacterial artificial chromosomes and fosmids^6,7^. The genome was updated in 2016 with data generated from Pacific Biosciences (PacBio) Single Molecule Real-Time (SMRT) sequencing and optical mapping to produce an ~88 Mb genome made up of 197 scaffolds, 205 contigs^8^. While still draft, the updated genome is highly contiguous and complete, with an N50 greater than 14 Mb and BUSCO and CEMGA estimates higher than 95%. This reference genome provides a foundation to assess the quality of genome assemblies generated with other platforms and software.

Single molecule sequencing platforms, such as the Oxford Nanopore Technologies (ONT) Minion and PacBio SMRT sequencing have drastically reduced the cost and required infrastructure for generating more complete and highly contiguous genome assemblies^9^. Multiple eukaryotic genomes have been successfully sequenced and assembled with reads generated from ONT platforms, including the human genome^10–13^. For example, Tyson *et al*. used reads generated from the ONT Minion to resolve complex genomic rearrangements and extended the genome of *Caenorhabditis elegans*^14^. That study demonstrated the potential of this technology to reconstruct and improve a high-quality nematode genome. We therefore sought to assess the performance of the ONT MinION platform for sequencing filarial parasitic nematodes. Using data generated from a single flowcell, we were able to *de novo* assemble a nearly complete nuclear genome of *B. malayi* that approached the quality of the reference genome in terms of size, contiguity, and content. The complete mitochondrial genome of *B. malayi* was assembled and obtained from individual reads, and the nearly complete genome of the *Wolbachia* endosymbiont (wBm) was also assembled. This study demonstrates the ONT MinION platform is able to generate the data necessary to assemble a nearly complete genome from filarial parasites using demonstrably fewer resources than previous approaches.

## Materials and Methods

### DNA extraction of parasite material

Adult *B. malayi* worms (FR3 strain) were provided by the NIH/NIAID Filariasis Research Reagent Resource Center for distribution by BEI Resources, NIAID, NIH^15^. Worms were kept in culture for 21 days prior to being frozen at −80C for storage. Three mature female worms were pooled in a 1.5 mL tube and crushed with a disposable pestle (VWR, Pennsylvania, USA). Disrupted worm tissue was incubated with 180 µL of Buffer ATL (Qiagen, Hilden, Germany) and 20 µL proteinase K (Qiagen) for 48 hours in a 56 °C water bath with occasional vortexing. Following incubation, DNA was extracted using the MagAttract HMW DNA kit (Qiagen) according to the manufacturer’s protocols. Total genomic DNA was eluted into 100 µL of 10 mM tris-HCL. A general size distribution of total DNA was determined using a 0.8% agarose gel. DNA quality and quantity were determined using a Nanodrop spectrophotometer (ThermoFisher, Massachusetts, USA) and Qubit fluorometer (ThermoFisher), respectively. Genomic DNA was subjected to size selection using 0.4x volume of AmpureXP beads (Beckman Coulter, California, USA) to remove small fragments, resulting in a lower concentration of DNA.

### Library preparation and sequencing

A total of 1.2ug of DNA was used as input for the 1D genomic ligation (SQK-LSK109) library preparation kit (ONT, Oxford, United Kingdom). The ligation sequencing kit was used to improve data yields. Libraries were prepared according to manufacturer’s protocols. Reaction volumes for the DNA repair, end-prep, and adaptor ligation steps were halved to conserve reagents. The final library quantity was 672 ng, and a total of 292 ng was used to load onto the sequencer. Based on the estimated size distribution, 25–40 fmol of library was sequenced using the ONT MinION Mk1B platform with a R9.4.1 flowcell. Prior to initiation, a total of 1,190 pores were available for sequencing per the initial mux scan. MinKNOW software (version 18.12.9, ONT) was used to run the flowcell with active channel selection every 1.5 hours and no script modifications. The flowcell was “refueled” at 24 hours by removing excess liquid from the waste chamber, opening both the priming port and SpotON sample port, and adding a mixture of 37.5 µL of SQB and 37.5 µL of H_2_O directly to the SpotON sample port. The sequencer was run for an additional 24 hours for a 48-hour run time. Raw.fast5 files were directly exported to an external SSD drive.

### Basecalling, genome assembly, and read alignments

Signal data (.fast5 files) was basecalled using Guppy (version 2.3.5, ONT) following completion of the sequencing run. The resulting.fastq files were used as input to generate run statistics with NanoPlot (version 1.19.0)^16^. Multiple approaches were used to generate a total of 4 genome assemblies. Reads were *de novo* assembled using Canu (version 1.7.1) and wtdbg2 (version 2.3) using default parameters^17,18^. Assemblies were created with Canu using 1) all reads generated, 2) a subset of total reads selected for size and quality using Filtlong (version 0.2.0)^19^, as well as with wtdbg2 using 3) all reads generated, and 4) the subset of reads >5 Kb in length. Following genome assembly, individual reads were aligned to the *B. malayi* reference genome (BioProject PRJNA10729) using minimap2 (version 2.15, -ax map-ont option) to determine depth of coverage across the genome as well as individual read nucleotide identity^20^. Depth of coverage was determined using samtools depth, and nucleotide identity was determined using read_length_identity.py script from Wick *et al*.^21,22^. Analysis software was run on a single node on the McDonnell Genome Institute high performance computing clusters. A typical node consists of dual CPUs: Intel(R) Xeon(R) CPU E5-2680 v4 (2.40 GHz) hosted on PowerEdge M630 blade servers.

### Nuclear genome assembly assessment

For the purpose of this study, all assemblies were compared to the *B. malayi* reference genome (obtained from parasite.wormBase.org in March, 2019). QUAST (version 5.0.2) and MUMmer (version 4.0.0) were used to assess assemblies for quality and relatedness^23,24^. BUSCO analysis was performed to assess the completeness of the MinION based assemblies and reference assembly (version 3.0.1)^25,26^. A selection of RNA-Seq samples were downloaded from EBI’s Array Express (https://www.ebi.ac.uk/arrayexpress/) and were subsequently cleaned using Trimmomatic (version 0.36)^27,28^. Cleaned reads were mapped to the MinION generated assemblies using HiSat2 (version 2.1.0) and the percentage of reads aligned was used to assess assembly completeness^29^. Genome polishing was performed with signal data (.fast5 files) using Nanopolish consensus (version 0.11.0) with default parameters^30^. Nanopolish was run with parallel in 50Kb segments of the genome and took around 3 days to complete using the compute resources stated above^31^. Percentage of coverage and identity was determined using MUMmer dna-diff. The polished genome was aligned to the reference genome using minimap2 (-ax asm5 option) and BEDtools (version 2.27.1) was used to query the alignment for areas of no coverage in 10Kb windows^32^. Because the reference genome has multiple scaffolds, which are represented by stretches of N’s, N’s were removed to calculate the percentage of length covered between the assemblies. Additionally, BEDtools was used to identify uncovered regions and intersect those regions with *B. malayi* features as another metric for assembly completeness.

### Mitochondria and Wolbachia genome assembly and assessment

The mitochondrial genomes were compared in Geneious (version 2019.0.3) using the Mauve plugin^33,34^. Nucleotide identity of individual reads corresponding to the mitochondrial genome was calculated as described above. The “Annotate from” tool in Geneious was used to transfer gene, tRNA, and rRNA annotations from the reference mitochondrial genome to the mitochondrial assembly. Nanopore reads aligning to the *Wolbachia* contig “wBm_Wolbachia” in the reference genome were extracted with samtools and used as input for *de novo* assembly using wtdbg2 as described above. Nanopolish was used to polish the resulting genomes as described above. The polished genome was compared to the reference *Wolbachia* genome using MUMmer dna-diff.

## Results

### Sequencing statistics

**Figure 1:**
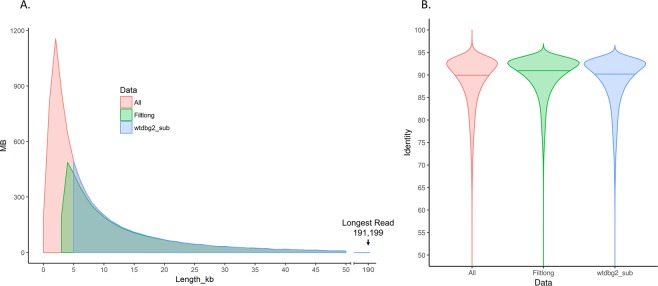
Length and identity of reads generated from a single MinION flowcell. **(a)** Read length distribution of all reads (red), reads filtered and subsampled with Filtlong (green), and reads filtered with a hard 5Kb cutoff (blue). Total number of reads were binned into 1Kb sets using BBMap and graphed using the geom_area function in the ggplot2 library in R. (**b)** Violin plots showing percent nucleotide identities of the same iterations of the data compared to the reference *B. malayi* genome. Percent identities of individual reads were determined using read_length_identity.py script (see Methods) and graphed using the geom_violin function in the ggplot2 library in R^55^.

All data presented in this study were generated from a single sequencing library on a single MinION flowcell. More than 2 million reads were generated that resulted in 7.7 Gb of called reads and an estimated 87.3X depth of coverage (Table 1, Supplementary Fig. S1). The mean and median read lengths were 3,594 and 2,303 nucleotides (n.t.), respectively, with an average read identity of 78.5% compared to the reference genome (Fig. 1). This average uses reads that did not align and resulted in an identity of value zero. The longest read produced by the sequencing run (191,199 n.t.) aligned to the reference genome with 81.6% identity. Data was filtered and subsampled to between 45–50X coverage to determine if using only the longest, highest quality reads for assembly would facilitate improved genome contiguity and shorten assembly time. Filtered data using Filtlong and a hard 5Kb cut-off increased both median (6,235 n.t., 7,841 n.t.) and mean read length (8,766 n.t., 10,641 n.t.), as well as average read identity (89.3%, 85.7%) compared to the reference genome (Fig. 1).

**Table 1:** Information on input sequences used for assemblies.

Read Set	Reads	Total Bases (Gb)	Median Read Length (Kb)	Median Q Score	Average Identity (%)	Estimated DOC^a^
All Reads	2,143,662	7.77	2.30	11.0	78.5	87.3
Filtlong Reads	513,325	4.50	6.23	10.3	89.3	51.0
Reads > 5 k	378,033	4.02	7.84	10.0	85.7	45.6

^a^Depth of Coverage.

### Subsampled read inputs produce more contiguous assemblies

**Figure 2:**
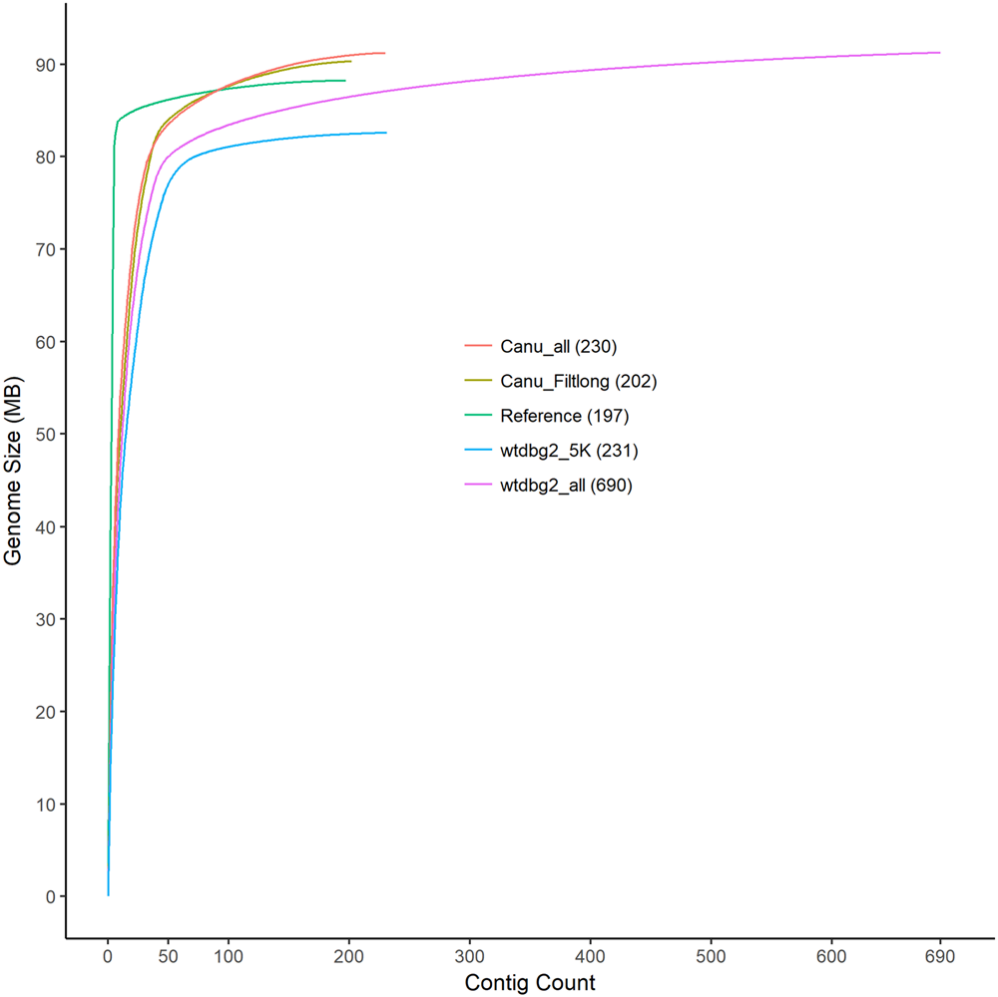
Cumulative lengths of all assemblies compared to the *B. malayi* reference genome. The Canu_Filtlong assembly (yellow line) most closely matched the reference assembly (green line) in length (89.7 Mb) with the fewest number of total contigs (202). The total number of contigs for each assembly is listed in parenthesis in the legend. Cumulative lengths graph was generated using the geom_line function in the ggplot2 library in R.

Four assemblies were generated with two tools, Canu and wtdbg2, using various iterations of the data generated (Table 2). The assembly time of wtdbg2 was remarkably faster than that of Canu. Allocating the same computational resources, all assemblies with wtdbg2 took hours on wall clock time, while the wall clock time for Canu took days. All generated assemblies have a GC content of ~28% that is similar to the reference assembly (28.5%). The total estimated genome size varied more in wtdbg2 assemblies compared to Canu assemblies. Similarly, total contig counts were more variable in wtdbg2 assemblies compared to Canu assemblies (Table 2, Fig. 2). For both assemblers, the use of filtered and subsampled data improved contiguity. However, the assembly generated with wtdbg2 using these data produced a genome that was substantially smaller (82.6 Mb) than the reference genome (88.2 Mb). This phenomenon is described in the wtdbg2 manual. The assembly generated with Canu using filtered and subsampled data was similar to the reference genome in size (89.7 Mb to 88.2 Mb) and contiguity (202 to 205 contigs). The Canu_Filtlong assembly had the highest percentage of RNA-seq reads aligning to the genome (78.5%) and BUSCO score (94.4% of expected gene content) compared to the other MinION assemblies. Therefore, the Canu_Filtlong assembly was used for additional analysis.

**Table 2:** Assembly metrics from four assemblies generated with MinION data.

Assembly Name	Size (Mb)	Number of contigs	Largest contig (Mb)	N50 (Mb) (#contigs)	N90 (Kb) (#contigs)	GC%	RNAseq mapping (%)	BUSCO (%)
Canu All	91.2	230	9.8	3.1 (8)	211.6 (42)	28.6	69.2	93.7
**Canu_Filtlong**	**89.7**	**202**	**10.7**	**2.4 (8)**	**400.5 (38)**	**28.6**	**78.5**	**94.4**
wtdbg2 All	91.3	690	9.0	2.4 (10)	64.1 (75)	28.4	73.3	92.1
wtdbg2 Reads >5 k	82.6	231	8.2	2.5 (10)	481.3 (43)	28.5	66.7	91.7
Reference (FR3)^a^	88.2	197	24.9	14.2 (3)	13,500 (5)	28.3	82.9	97.0

^a^The reference genome (BioProject:PRJNA10729) contains both scaffolds and contigs, here we are listing the number for scaffolds.

### Genome assembly using nanopore data resembles reference assembly

**Figure 3:**
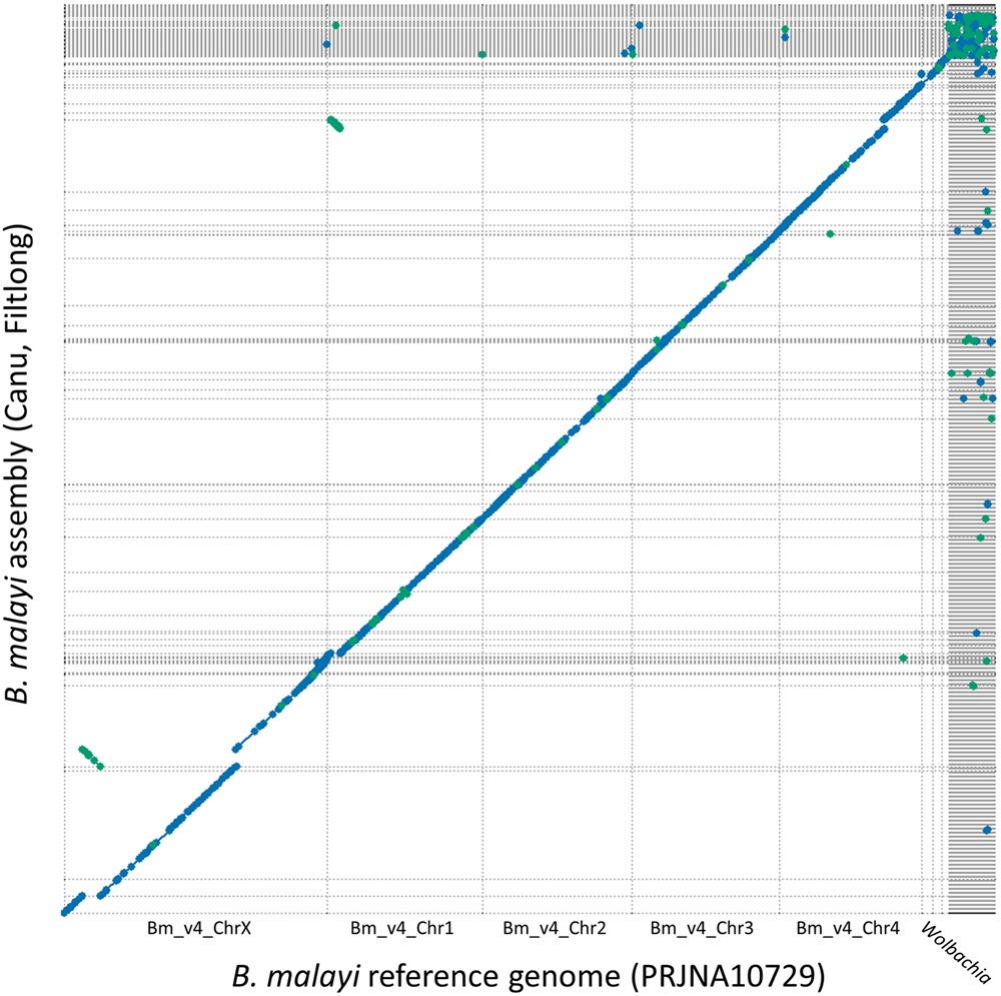
Alignment of the Canu_Filtlong assembly to the *B. malayi* reference genome shows a high degree of similarity. Canu_Filtlong assembly (y-axis) spans the 5 major contigs/scaffolds that make up >93% of the *B. malayi* reference genome and the wBM genome (x-axis). The majority of disagreements between the assemblies occur in smaller contigs as seen by the dots in the lattice of vertical and horizontal lines in the upper right corner of the graph. Forward matches are displayed in blue, reverse matches are displayed in green. The dotplot representing a 1-to-1 alignment of the two assemblies was generated with the 0.1 delta file output from dnadiff using mummerplot with options -fat -png -filter -medium options selected in the MUMmer package.

**Figure 4:**
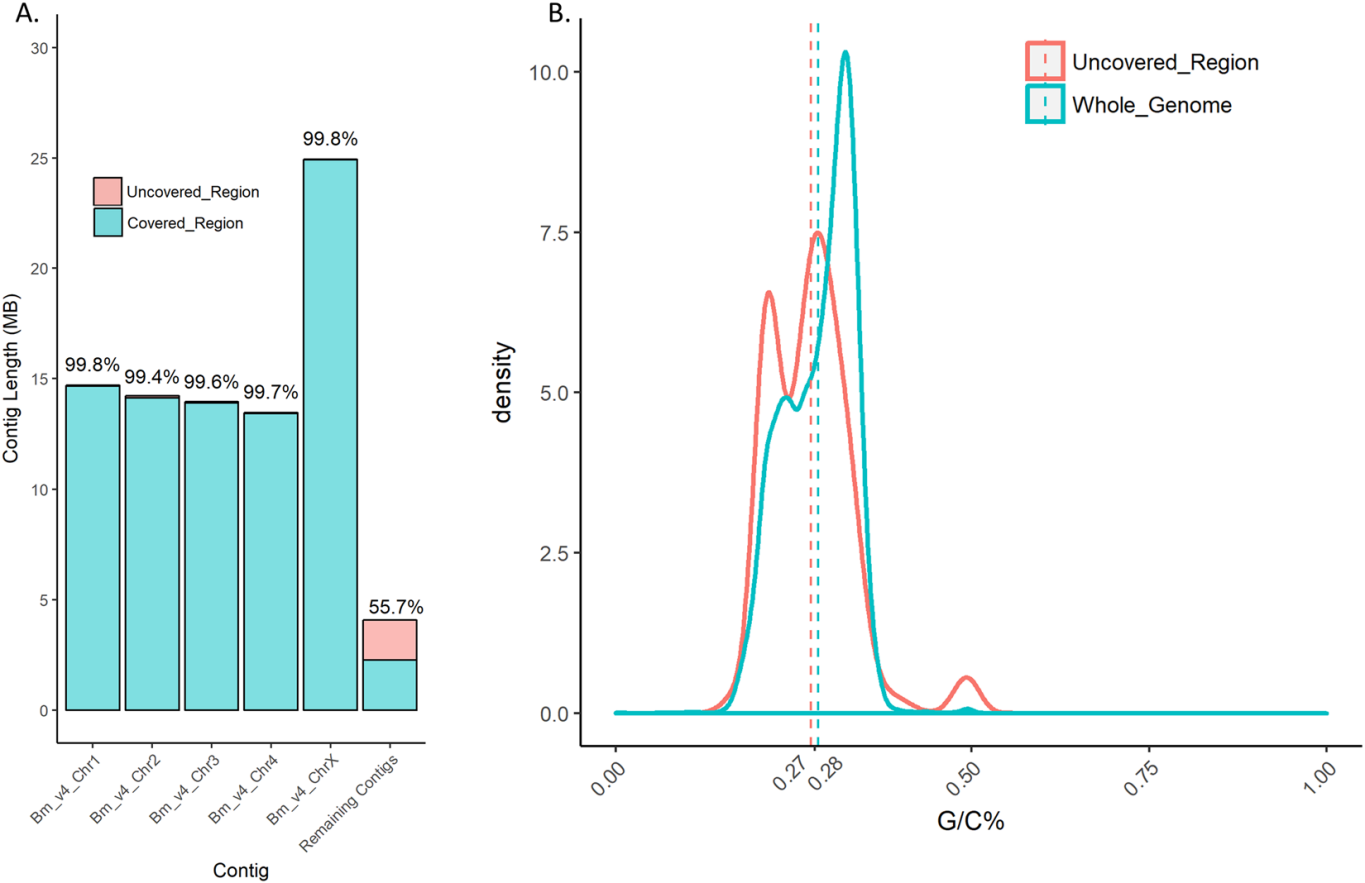
The Canu_Fitlong assembly covers greater than 99% of the 5 major scaffolds/contigs in the reference genome. (**a**) Bar graph showing the percent coverage of the 5 major contigs/scaffolds and all the remaining contigs combined. Number above each bar is the percent of total coverage. Alignments of the assemblies were made with minimap2 -ax asm5 and coverage was determined in 10 Kb windows across the reference genome with samtools depth and BBMap. Stretches of N’s used for scaffolding were removed in percentage calculations. The bar graph was generated with geom_bar function in the ggplot2 library in R. (**b)** Density plot showing the %GC content of the whole genome (blue) and the uncovered regions of the genome orange with dotted lines representing the average GC content. GC content was calculated using BBMap stats.sh. Density plot was generated using geom_density function in the ggplot2 library in R.

We employed a variety of methods to compare the MinION generated assembly to the reference *B. malayi* assembly. First, the assemblies were aligned, compared, and visualized using MUMmer (Fig. 3). The two genomes agree across the 5 major contigs of the reference genome, although there are likely portions of the Canu_Filtlong genome that are assembled incorrectly. For example, a 1.77 Mb portion of a 8.1 Mb contig is inverted and aligns to a different region on Chromosome X, and a 0.9 Mb portion of a 7.1 Mb contig is inverted and aligns to Chromosome 1, where the remainder of the contig makes up a large portion of Chromosome 4 (Fig. 3). In total, 96.9% of the reference genome was covered by the Canu_Filtlong assembly. A total of 93.3% of the reference genome is contained in 4 scaffolds and 1 contig, of which the Canu_Filtlong assembly spanned greater than 99% (Figs. 3 and 4a). The remaining 3.1% of the genome that was not covered by the Canu_Filtlong assembly was largely the result of no alignments to the smaller contigs that were not placed within the larger reference assembly (Figs. 3 and 4a). A single 3,020 b.p. contig was produced in the Canu_Filtlong assembly that was not present in the reference genome. A BLASTn query of this contig produced a top hit to Homo sapiens isolate 1a satellite DYZ1 sequence (Genbank I.D. KF941193.1), indicating likely contamination with human DNA. A total of 190 gene features (0.9% of total coding genes) fell within the 3.1% of the genome not covered by the Canu_Filtlong assembly (Supplementary Data S1). There were no gene features identified in areas of no coverage based on individual reads, as most regions not covered by reads were where contigs are scaffolded (i.e. stretches of N’s). For the reference genome, the GC content of the uncovered contigs (27%) did not differ substantially from the GC content of the whole genome (28%) (Fig. 4b).

Additional analyses were performed to assess and quantify the mismatches between the two assemblies. The average identity of the MinION-derived assembly across the reference nuclear genome was 98.6%. A substantial portion of mismatches identified were insertions/deletions (indels) (Table 3). Following genome polishing with Nanopolish, the overall nucleotide identity between the two assemblies increased to 99.2%. While the number of single nucleotide polymorphisms (SNP) decreased by 5,711 (6.5% decrease), the number of indels were reduced by 487,619 (46.8% decrease). Polishing the genome resulted in 43% less mismatches when compared to the original assembly. The number of mismatches were evenly distributed across the 5 major scaffolds/contigs and the remaining contigs, as measured by similar mismatches per 1,000 b.p. (Supplementary Table S1).

**Table 3:** Results of assembly polishing using Nanopolish.

Metric	Pre-Polishing	Post-Polishing
Length (Mb)	89.7	90.3
Reference Covered (%)	96.1	96.9
Average Identity (%)	98.6	99.2
GC Content (%)	28.6	28.6
Total SNPS	87,961	82,250
Total Indels	1,041,371	553,752
Total Mismatches	1,129,332	636,002

We sought to determine if these mismatches would impact the identification of gene sets and their effect on the coding sequences of identified genes. BUSCO assessment of the polished Canu_Filtlong assembly identified 95.7% of expected single-copy orthologs, while 97.0% were identified in the reference assembly (Supplementary Table S2). Of the genes identified in the polished Canu_FIltlong assembly, 3.8% were fragmented, compared to 0.3% in the reference assembly, indicating more genes were only partially recovered. The average CDS length was substantially shorter in the polished Canu_Filtlong assembly compared to the reference, 120 b.p. vs. 133.7 b.p, respectively, suggesting the introduction of premature stop codons and frame shift mutations in coding sequences (CDS) (Supplementary Table S2). As well, the average number of CDS per gene was higher for the polished Canu_Filtlong assembly.

### Assessment of the Brugia malayi mitochondrial genome

Individual reads aligned to the mitochondrial genome with greater than 500X coverage (Supplementary Fig. S1). No contig in the final assembly produced a high-quality alignment to the mitochondrial genome. However, a 13,264 b.p. sequence was identified in the “unitig” output of Canu that aligned to the reference mitochondrial genome with 96% nucleotide pairwise identity. Each of the 12 genes, 2 rRNA subunits, and 22 tRNA regions identified in the annotated reference genome were found on the Canu_Filtlong assembly of the mitochondrial genome. Due to the small size of the mitochondrial genome, we sought to determine whether individual reads spanned it’s the entire length. Aligned reads were filtered by length to correspond to >90% (12,291b.p.) of the mitochondrial genome length. 201 reads between 12,291-13,647b.p. in length aligned to the mitochondrial genome with an average nucleotide identity of 95% (Supplementary Fig. S2).

### Assembly of the Wolbachia endosymbiont wBM

**Figure 5:**
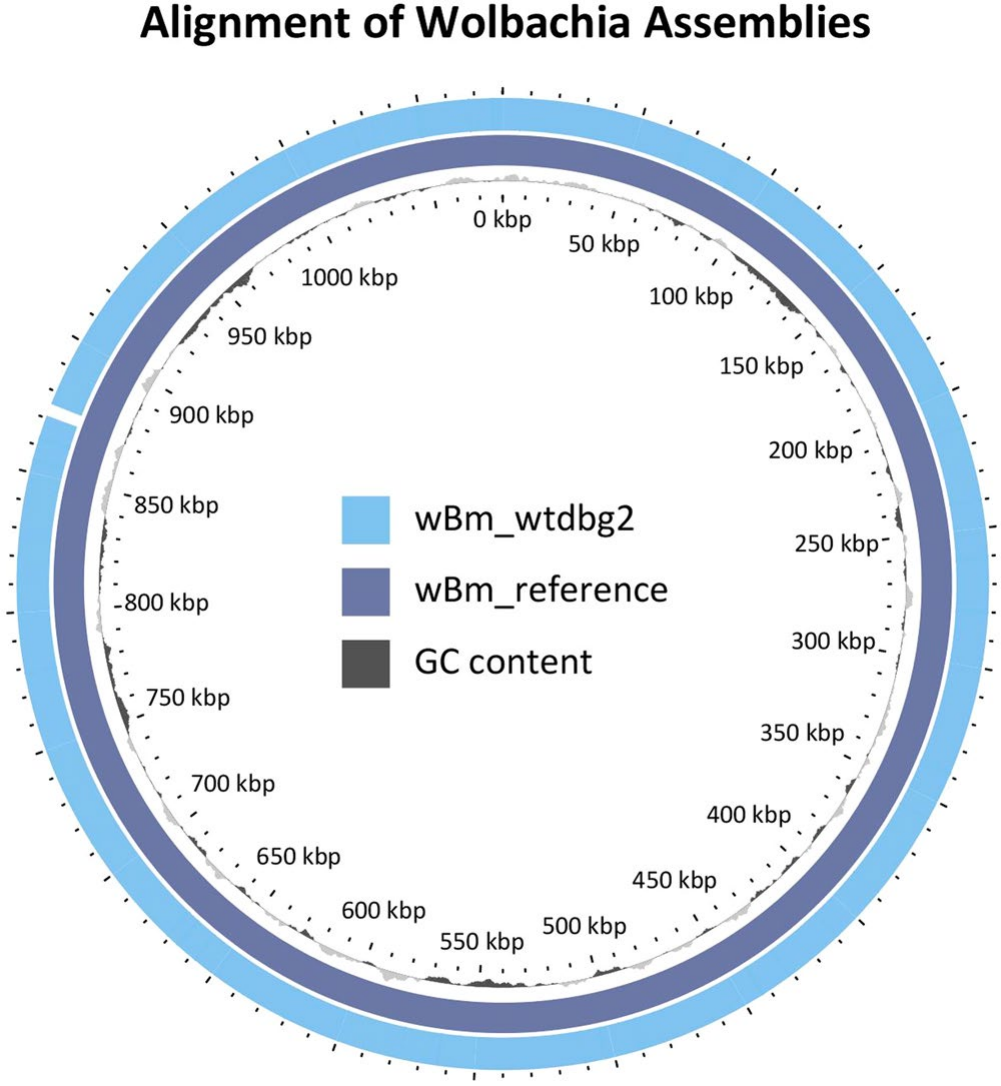
The genome of the *Wolbachia* endosymbiont assembled into a single contig. The outside light blue track represents the *Wolbachia* assembly using only ONT data. The inner dark purple track represents the reference *Wolbachia* genome. The light and dark grey track represents AT and GC content, respectively. A single insertion of 4.3 kb at 870 kb was not represented in the ONT assembly. *Wolbachia* genomes were aligned using MUMmer and visualized using Gview^56^.

In addition to the nuclear and mitochondrial genomes of *B. malayi*, most of the ~1 Mb genome of wBM, a bacterial endosymbiont of *B. malayi*, was assembled into two contigs that spanned 96.6% of the reference wBM genome with 99.3% nucleotide identity. However, a large gap (~30 Kb) of the reference wBM genome was not covered by the ONT assembly, although it was covered by individual reads. Using these reads as input into wtdbg2, a single contig 1,071,092 b.p. in length was generated, and the ~30 Kb gap was reduced to 4.3Kb (Fig. 5). Following genome polishing with Nanopolish, the genome generated with only *Wolbachia* reads aligned to the reference wBM genome with 99.5% average identity.

## Discussion

Advances in long-read sequencing technologies are drastically reducing the cost of generating high-quality *de novo* genome assemblies. Reference genome assemblies for many parasitic worm species are still largely fragmented^35,36^. In this study we demonstrated that data from a single ONT MinION flowcell was sufficient to assemble a contiguous and nearly complete genome of the filarial parasite *B. malayi*, the *B. malayi* mitochondria genome, and a nearly complete *Wolbachia* genome. However, the MinION-derived assembly resulted in shorter N50 and N90 lengths, as well as lower BUSCO percentages and shorter CDS when compared to the reference genome assembly from the same strain of worm. Although this assembly is not as complete as the reference, it was generated with fewer resources. An immediate benefit of the ONT MinION technology in parasite genomics will be to a) improve current draft genome assemblies and b) quickly generate novel reference genomes for worm species that currently lack genome information. While data from a single flowcell was sufficient for the assembly of the moderately sized *B. malayi* nuclear and mitochondrial genome, many parasitic worm genomes are hundreds of millions of nucleotides, thus proportionally increasing the amount of data needed to generate a high-quality assembly.

As generating data for genome sequencing and assembly projects are expensive with conventional approaches, it is important to note that the total cost of equipment and consumables (in addition to common laboratory reagents) for the data generation portion of this project was less than $1,000 U.S. However, the computational costs of *de novo* assembly of a moderately sized genome are steep. For this study, we assembled the *B. malayi* genome using Canu on a remote server that contained substantially more compute resources than typically found on a personal computer, although assembly programs can be operated on less powerful machines. As well, the MinION platforms output an additional file type (.fast5) not seen on other sequencing platforms, increasing the amount of storage space required for large-scale sequencing projects. The ONT MinION platform has the potential to rapidly expand our understanding of the genomes for many neglected tropical parasites, however data storage and computational cost may still be prohibitively high for some groups who do not have access to compute resources. Third-party companies are increasing access to both external data storage and compute resources that can be leveraged to perform bioinformatic analysis.

In addition to generating and improving upon reference genomes, the MinION platform can facilitate genomic investigations of disease transmission. Indeed, the MinION has been at the center of multiple outbreak investigations including the West African Ebola virus epidemic and the Zika virus epidemic in the Americas^37,38^. Genomic studies of filarial parasites may provide useful epidemiological insights into worm transmission dynamics and local population structure. In the context of the GPELF, which uses MDA of anti-helminthic drugs to interrupt parasite transmission, fine-scale genomic studies might identify sources of residual transmission in communities following MDA. While MDA has proven effective at shrinking the map of lymphatic filariasis endemic areas, residual transmission following multiple rounds of MDA has been documented in multiple countries^39–47^. A comparative genomic approach may help elucidate the dynamics of residual transmission by identifying population specific markers to determine whether reinfection, reintroduction, or incomplete clearance has occurred following MDA^48–50^. While the ONT MinION has the potential to bring a genomics component to a large-scale disease elimination program such as the GPELF, technical hurdles will need to be overcome.

For instance, the sample type used in this study, whole adult worms, are typically not accessible in patients with filariasis as they reside in “nests” within the lymphatic system^4^. Nests can be detected by ultrasound but can only be extracted by invasive surgery. Microfilariae that circulate in the blood can be obtained by finger prick as is routinely done for diagnostic purposes. However, microfilariae are much smaller than adult worms and are targeted by the anti-parasitic drugs given during MDA. This decreases the amount of parasite material available for obtaining high molecular weight DNA required for MinION sequencing. We were able to obtain over 1 µg of DNA from three adult worms, while thousands of microfilariae would be needed to generate a similar quantity of DNA. Contamination with host DNA also becomes problematic when trying to sequence DNA isolated from circulating microfilariae. This can be improved by filtration of microfilariae from peripheral blood or differential lysis and purification techniques for manual separation of parasite from host material. As well, molecular techniques like whole genome amplification would work to increase the signal to noise ratio in complex biological samples, although they do introduce replication errors. Small *et al*. overcame this issue by sequencing individual L3 larvae collected from mosquitoes that fed on blood from an infected individual to shed light on intrahost population dynamics^48^. However, to discern transmission dynamics within a community, sequences would need to be obtained from many parasites, ideally from samples collected during routine monitoring and evaluation programs. Recent studies have shown the benefit of optimizing DNA extraction techniques to generate whole genome sequences from a minute amount of parasite material^51^. Continuing to improve techniques for concentrating, purifying, and extracting DNA from worms in peripheral blood will be necessary to provide adequate input for MinION sequencing.

The FR3 strain of *B. malayi* sequenced in this study is highly inbred and likely has little genetic variation. The accuracy of our consensus assembly following polishing compared to the reference was greater than 99%, and most of the identified mismatches were indels. Indels in homopolymer regions are the most common error type encountered on the ONT MinION platform, which implies that the majority of the mismatches are the result of sequencing errors as opposed to genuine variation^52,53^. These errors likely resulted in premature stop codons in the coding sequence of genes, as indicated through shorter average CDS lengths and an increase in the average number of CDS predicted per gene. This error rate may still be too high to confidently detect informative variants in a population. Further complicating matters, individuals with lymphatic filariasis are typically infected with more than one genetically distinct worm^49^. Increasing sequencing depth to parse out specific haplotypes could overcome this problem. We obtained between 70–100x coverage across most of the genome with a single flowcell. However, this result was obtained with ideal input material from laboratory-controlled infections. As well, filtered and subsampled data with preference given to the longest reads produced the best assemblies. These results suggest that it may be possible to obtain sufficient sequencing depth by multiplexing samples on a single flow-cell. Because multiple loci are needed to accurately represent population structures, whole genome approaches will be needed to identify informative markers^51,54^. However, at its current state, the platform would not be recommended for population genomic studies of filarial worms.

This study demonstrates the MinION’s ability to generate adequate data to *de novo* assemble the genome of a eukaryotic parasite at minimal cost. The ONT platforms allow for genomic analysis of understudied organisms that were previously cost prohibitive, as well as in areas that did not have the infrastructure or capacity for genome sequencing. As the accuracy and ease of use of the MinION continues to improve, it may be feasible to include genomic analysis as a part of large-scale disease elimination programs.

## Supplementary information

Supplementary Information

Supplementary Dataset 1

## Data Availability

All sequencing data has been submitted to NCBI SRA database (BioProject: PRJNA565193) and the final assembly has been submitted to Nematode.net (www.nematode.net).
