# Supplementary material for: De novo Assembly of the Brugia malayi Genome Using Long Reads from a Single MinION Flowcell
Source: Sci Rep. 2019 Dec 20;9:19521. doi: 10.1038/s41598-019-55908-y (PMC6925183; doi:10.1038/s41598-019-55908-y)
Supplement: Supplementary file 1 — Supplementary Information [file 41598_2019_55908_MOESM1_ESM.pdf]

Supplementary information

Title: *De novo* Assembly of the *Brugia malayi* Genome Using Long Reads from a Single MinION Flowcell

Joseph R. Fauver[1,#,*], John Martin[2], Gary J. Weil[1], Makedonka Mitreva[1,2] Peter U. Fischer[1]

1- Division of Infectious Diseases, Department of Medicine, Washington University School of Medicine, St. Louis, MO, United States

2- McDonnell Genome Institute, Washington University School of Medicine, St. Louis, MO, United States

# Current address: Department of Epidemiology of Microbial Diseases, Yale School of Public Health, New Haven, CT, United States

*Correspondence to Joseph R. Fauver fauverjoseph@gmail.com

Supplementary Figure 1- Depth of coverage across each major contig in the reference genome, mitochondrial genome, and wBM genome.

Supplementary Figure 2- Percent nucleotide identity of reads covering greater than 90% of the mitochondrial genome

Supplementary File 1- Gene features identified in areas of no coverage in the reference genome.

Supplementary Figure 1

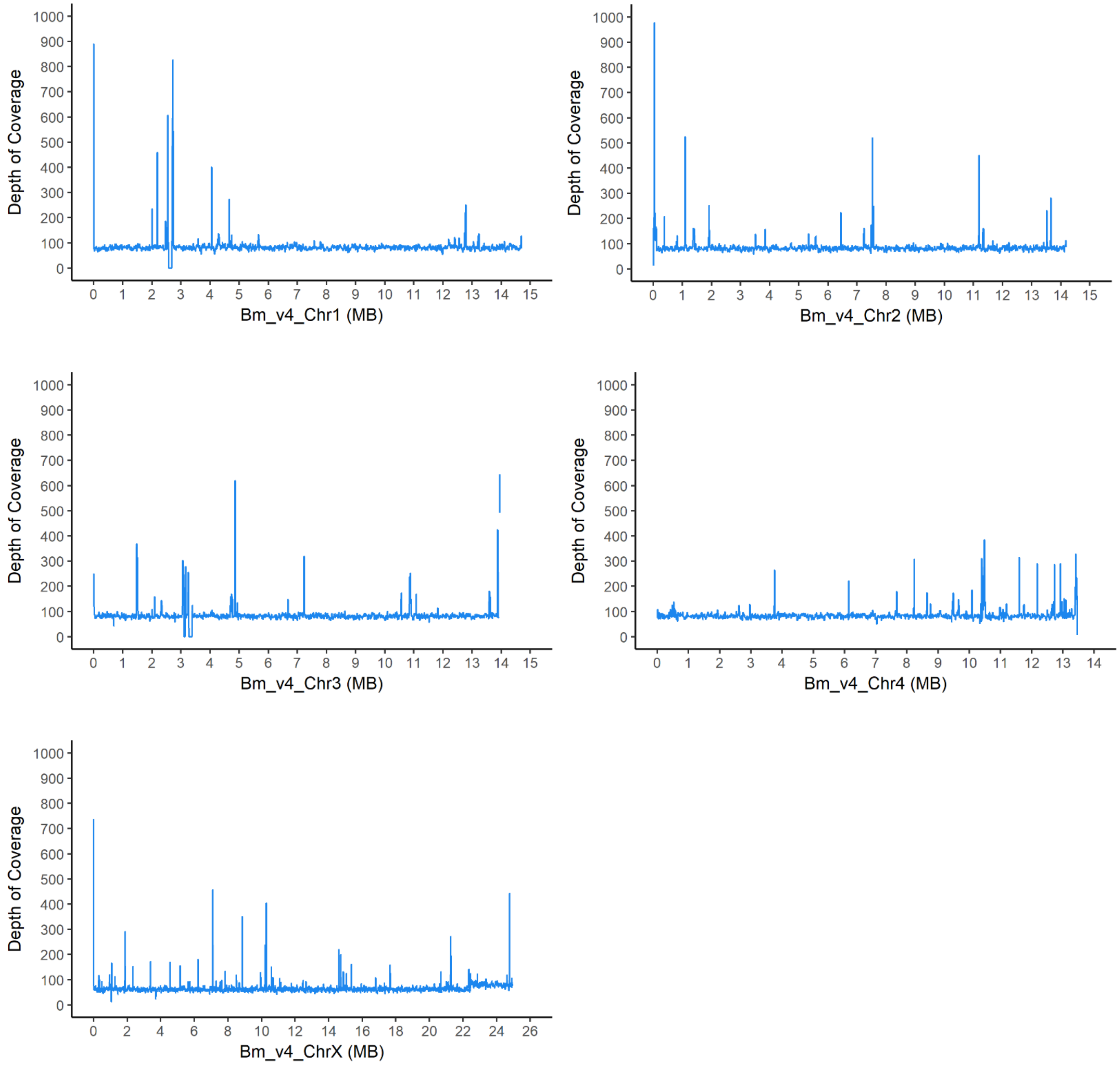

Supplementary Figure 1 continued

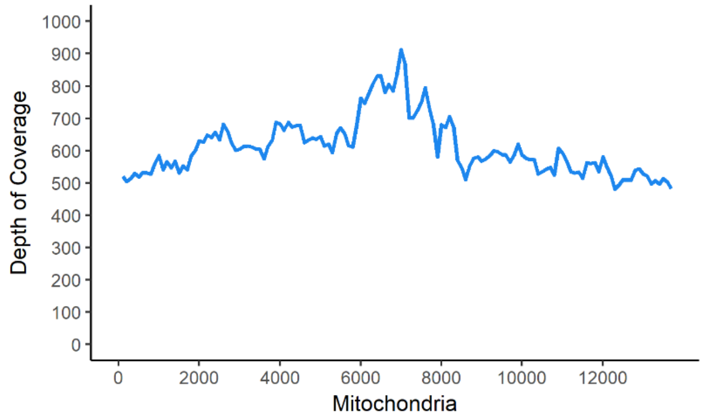

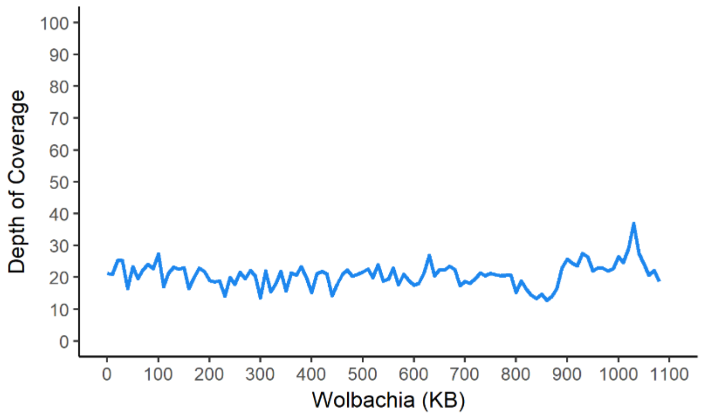

Supplementary Figure 2

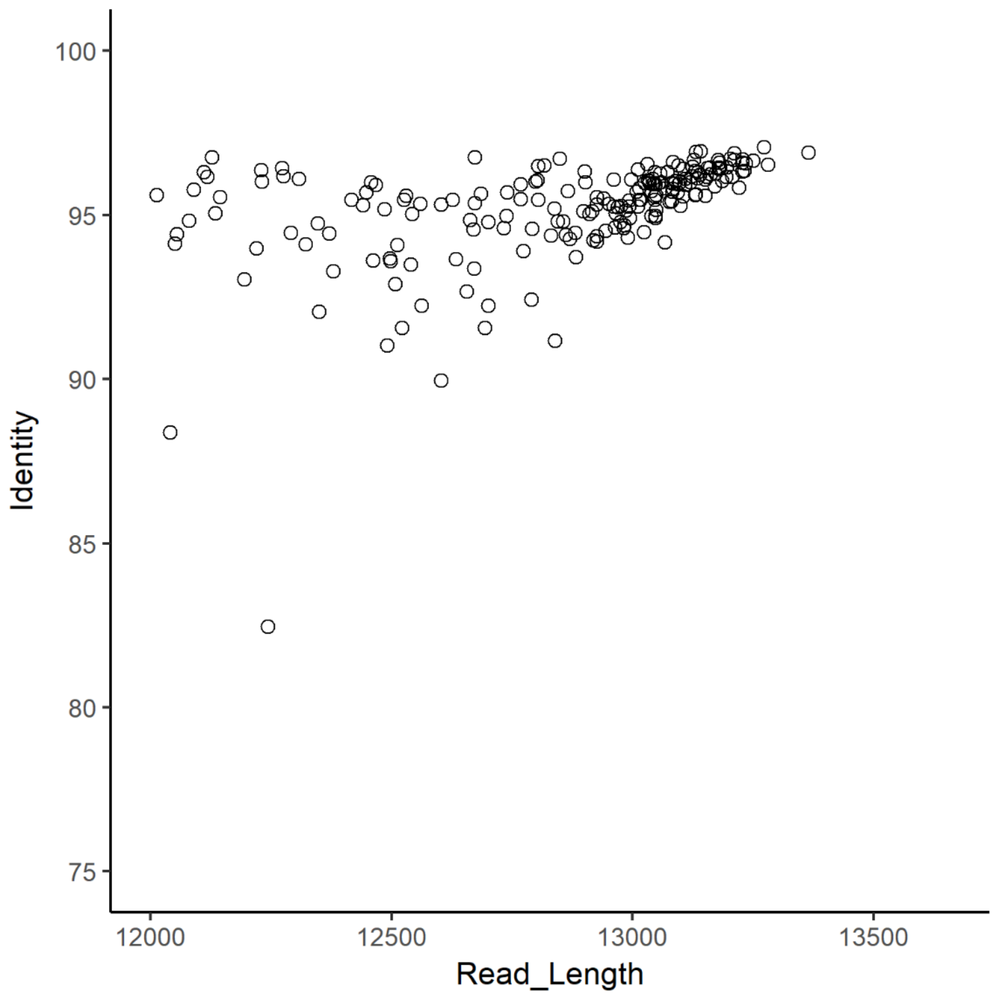

Supplementary Table 1: Assembly mismatches compared to the reference genome							
		ONT Assembly Pre-Polishing			ONT Assembly Post-Polishing		
**Genome Segment**	**Length (b.p.)**	**Total Mismatches**	**Mismatches per b.p.**	**Mismatches per 1kb**	**Total Mismatches**	**Mismatches per b.p.**	**Mismatches per 1kb**
Bm_v4_Chr1	14701151	198499	0.01	13.50	112895	0.008	7.68
Bm_v4_Chrm2	14214749	195689	0.01	13.77	111435	0.008	7.84
Bm_v4_Chr3	13951302	188196	0.01	13.49	103327	0.007	7.41
Bm_v4_Chr4	13467244	194188	0.01	14.42	110512	0.008	8.21
Bm_v4_ChrX	24943668	279625	0.01	11.21	146124	0.006	5.86
Remaining contigs	5877599	65291	0.01	11.11	46229	0.008	7.87
5 Major Contigs	81278114	1056197	0.01	12.99	584293	0.007	7.19
Nuclear Genome	87155713	1121588	0.01	12.87	630522	0.007	7.23

Supplementary Table 2: BUSCO/CDS Statistics for Reference and MinION Assemblies

**BUSCOs (N=303)**	**Reference Assembly**	**Canu_Filtlong Assembly**	**Canu_Filtlong_Polished**
Complete and single-copy BUSCOs	289	262	277
Complete and duplicated BUSCOs	4	0	2
Fragmented BUSCOs	1	24	11
Missing BUSCOs	9	17	13
Total BUSCOs identified	294	286	290
Proportion of BUSCOs identified	97.00%	94.40%	95.71%
Avg CDS Length (b.p.) per shared BUSCO*	133.7	106.8	120
Avg Number of CDS per shared BUSCO	9.6	10.5	10.5

*- The assemblies shared a total of 160 complete single-copy BUSCOs
